# Collaborative Optimization of Model Pruning and Knowledge Distillation for Efficient and Lightweight Multi-Behavior Recognition in Piglets

**DOI:** 10.3390/ani15111563

**Published:** 2025-05-27

**Authors:** Yizhi Luo, Kai Lin, Zixuan Xiao, Yuankai Chen, Chen Yang, Deqin Xiao

**Affiliations:** 1 Key Laboratory of Agricultural Equipment Technology, College of Mathematics and Informatics, South China Agricultural University, Guangzhou 510642, China; luoyizhi@gdaas.cn (Y.L.); scau_kk@stu.scau.edu.cn (K.L.); ab9853211@stu.scau.edu.cn (Y.C.); 2 Institute of Facility Agriculture, Guangdong Academy of Agricultural Sciences, Guangzhou 510640, China; yangcheng@gdaas.cn; 3 State Key Laboratory of Swine and Poultry Breding Industry, Guangzhou 510640, China; 4 College of Engineering, South China Agricultural University, Guangzhou 510642, China; eiiinx@stu.scau.edu.cn

**Keywords:** piglet, multi-behavior recognition, prune, distill, precision livestock farming

## Abstract

In modern intensive pig farming, accurately monitoring piglet behavior is crucial for health management and improving production efficiency. However, the complexity of existing models demands high computational resources, limiting the application of piglet behavior recognition in farming environments. In this study, the piglet multi-behavior-recognition approach is divided into three stages. In the first stage, the LAMP pruning algorithm is used to prune and optimize redundant channels, resulting in the lightweight YOLOv8-Prune. In the second stage, based on YOLOv8, the AIFI module and the Gather–Distribute mechanism are incorporated, resulting in YOLOv8-GDA. In the third stage, using YOLOv8-GDA as the teacher model and YOLOv8-Prune as the student model, knowledge distillation is employed to further enhance detection accuracy, thus obtaining the YOLOv8-Piglet model, which strikes a balance between the detection accuracy and speed. Compared to the baseline model, YOLOv8-Piglet significantly reduces model complexity while improving detection performance, with a 6.3% increase in precision, 11.2% increase in recall, and an mAP@0.5 of 91.8%. The model was deployed on the NVIDIA Jetson Orin NX edge computing platform for the evaluation. The average inference time was reduced from 353.9 ms to 163.2 ms, resulting in a 53.8% reduction in the processing time. This study achieves a balance between model compression and recognition accuracy through the collaborative optimization of pruning and knowledge extraction.

## Simple Summary

In modern intensive pig farming, accurately identifying piglet behavior characteristics is a key strategy for improving health monitoring and animal welfare management in intensive farming systems. This study proposes the YOLOv8-Piglet lightweight architecture, designed to accurately recognize eight typical piglet behaviors. YOLOv8-Piglet integrates LAMP pruning and BCKD knowledge distillation to ensure real-time recognition performance while operating under limited computational resources. It offers a solution for precise behavior monitoring in piglet welfare management and contributes effectively to the intelligent transformation of the livestock industry.

## 1. Introduction

The growing global demand for meat, driven by population growth, has led to the expansion of pig farming. In the process of pig breeding, pig behavior relates to health, welfare, and the growth status, thereby indirectly influencing yields and economic benefits in the industry [1]. At the same time, the daily behavior of piglets is also an important indicator of their overall health and development [2]. Among these, lying provides necessary rest [3], while sitting and standing promote muscle development and mobility [4], collectively reflecting the overall health of piglets. Drinking and suckling are particularly critical, as subtle changes in these behaviors often serve as early warning signals of health issues [5]. For instance, when piglets are sick, they commonly exhibit symptoms such as decreased food intake [6]. Additionally, the social behavior of piglets also reflects their welfare [7]. Mounting behavior can increase in new or overcrowded environments, serving as an indicator to assess whether the pigs are in a comfortable state [8]. Head knocking and biting ears are aggressive behaviors in piglets that may result in stress, injuries, and economic losses [9]. The automated monitoring and analysis of piglet behaviors help to improve their health, welfare, and productivity [10], while also supporting sustainable pig farming [11]. However, regarding the complexity of farming environments (such as obstruction by barriers and complex indoor lighting) and the complexity of piglet behaviors (such as occlusion between piglets and the intricacy of social behaviors), the actual recognition process presents significant challenges for the detection model [12].

In early studies, traditional digital image processing and machine learning methods were widely used for pig behavior classification. Gronskyte et al. [13] applied optical flow methods to analyze slaughterhouse unloading videos, allowing the real-time monitoring of pig behavior and the detection of events like tripping or stepping. Nasirahmadi et al. [14] used binary images to calculate boundaries and convex hulls, followed by training a linear SVM classifier to detect lateral and sternal lying postures. However, traditional methods rely on manual feature extraction, which may affect their robustness and accuracy in dynamic and complex farming environments.

In recent years, the rapid development of deep learning has driven the advancement of precision agriculture. Deep learning methods can automatically extract features, providing substantial advantages across various tasks. Ji et al. [15] used an improved YOLOX to recognize pig postures, such as standing, lying, and sitting, achieving an mAP of 95.7% in overall posture recognition. Wang et al. [16] improved the YOLOv3 model by incorporating the Convolutional Block Attention Module (CBAM), enhancing the model’s ability to extract pig features. Li et al. [17] proposed an enhanced YOLOX model incorporating a normalization attention mechanism to effectively detect pig attack behaviors, achieving a precision rate of 93.21%. Mao et al. [18] developed the DM-GD-YOLO model, integrating multi-path coordinate attention and a gather-and-distribution mechanism to recognize both common and abnormal pig behaviors effectively, achieving an accuracy of 88.2%. However, the large parameter counts and computational complexity of these models present challenges for implementing piglet-behavior-recognition systems on devices with limited computing resources. To address these challenges, Wang et al. [19] proposed deploying large models on cloud servers, utilizing cloud resources and infrastructure to perform agricultural detection tasks that require substantial computational power. However, this method has several limitations: network transmission leads to delays [20], renting cloud servers incurs high costs [21], and there is a potential risk of agricultural data leakage [22]. Therefore, lightweight models designed for edge deployment provide a more secure and efficient alternative.

Recently, the trend of model lightweighting in smart agriculture has gained popularity. Liu et al. [23] introduced a lightweight RTMDet model for pig behavior detection, incorporating the MobileNetV3 backbone to significantly reduce model parameters. Gong et al. [24] proposed the GAB-YOLO model, integrating GhostNet and the Self-Attention Mixed Module as the backbone, reducing parameters by 14.5%. Luo et al. [25] proposed a piglet multi-behavior recognition model, PBR-YOLO, by integrating GhostNet as the backbone and an efficient multi-scale attention (EMA), achieving a 9.1 ms reduction in inference latency and a 59.1% decrease in the parameter count. Wang et al. [26] proposed a lightweight pig face detection method based on YOLOv8, utilizing a shared parameter detection head (SPHead), achieving a 17.0% reduction in parameters and a 38.2% decrease in computation. Although the above manually designed architectures have shown promising results in reducing computational and memory costs, they still face limitations in certain aspects. On one hand, manual design heavily depends on expert knowledge to balance accuracy, efficiency, and computing cost, which often leads to sub-optimal solutions [27]. On the other hand, designing and optimizing compact modules requires extensive experimentation and fine-tuning, increasing the development complexity and cost [28].

Moreover, the computational cost of the above lightweight networks is still relatively high, indicating potential for further compression and optimization [29]. Model compression reduces the network size by removing redundant parameters and structures. Model compression is divided into pruning, knowledge distillation, quantization, and low-rank decomposition [30]. Collaborative design and optimization is a promising direction that enables various compression methods to work together effectively [31]. To deploy the piglet multi-behavior recognition model on edge devices with limited computational resources in farming environments while maintaining a balance between detection performance and model lightweighting, this study proposes a three-stage collaborative optimization scheme. In the first stage, the LAMP pruning algorithm is used to eliminate non-essential redundancies, resulting in a lightweight YOLOv8-Prune model. In the second stage, the AIFI module and the Gather–Distribute mechanism are introduced based on YOLOv8, leading to the YOLOv8-GDA model. In the third stage, knowledge distillation is applied, with YOLOv8-GDA as the teacher model and YOLOv8-Prune as the student model, to further enhance the detection accuracy, resulting in the YOLOv8-Piglet model, which strikes a balance between detection accuracy and speed.

The main contributions of this study are as follows:

1. A comprehensive dataset was established, encompassing various piglet behaviors in an intensive farming environment, including complex environments and occlusion scenarios.
2. A collaborative optimization model, YOLO-Piglet, was proposed for piglet multi-behavior recognition, integrating pruning and distillation to achieve a balance between accuracy and model lightweighting.
3. TensorRT technology was employed to optimize and accelerate the model, allowing for efficient deployment and inference on the NVIDIA Jetson Orin NX edge device (Nvidia, Santa Clara, CA, USA).

The rest of the paper is organized as follows: Section 2 describes the dataset construction, model architecture, and the proposed improvements. Section 3 presents the experimental setup, evaluation metrics, and results. Section 4 discusses the findings in depth. Finally, Section 5 concludes the study with a summary and future directions.

## 2. Materials and Methods

### 2.1. Materials

**Figure 1:**
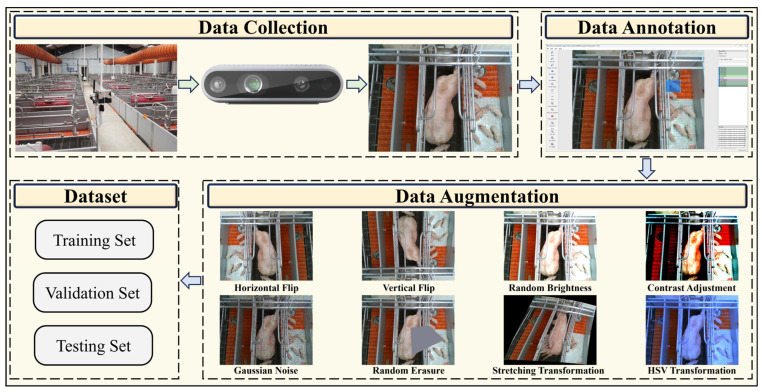
Dataset production process.

As shown in Figure 1, the main steps involved in data acquisition and processing in this study are as follows: (1) data collection, images of piglet behavior are captured using cameras installed above the pigpens; (2) data annotation, the collected images are annotated for behavior recognition; (3) data augmentation, various augmentation techniques, including flips, random brightness, and contrast adjustments, are applied to expand the dataset; (4) dataset division, the dataset is divided into training, validation, and testing sets.

#### 2.1.1. Pig Farm Environment

The dataset used in this study was collected from a standardized commercial pig farm located in Yunnan Province, Southwest China. As shown in the “Data Collection” section of Figure 1, the pigsty is designed with 12 rows, each divided into 12 pens, with a maximum capacity of 144 lactating sows. The pigsty is equipped with environmental control devices and a ventilation system to regulate the temperature and humidity inside, ensuring an optimal farming environment. The main structure of the pens is made of standard welded hot-dip galvanized steel pipes, with high-density polyethylene (HDPE) non-slip leakproof flooring and partitions made of polyvinyl chloride (PVC) panels. Each individual pen is equipped with a stainless-steel automatic feeding trough, nipple drinkers, and piglet feeding trays to ensure that both sows and piglets can feed and drink smoothly. Farm staff regularly replenish the feed in each gestation pen to ensure a continuous and sufficient food supply for the sows. After feeding, veterinarians conduct routine health checks, monitor the health status of the pigs in real-time, and promptly address any signs of abnormalities or illness.

#### 2.1.2. Data Collection

The dataset used in this study encompasses eight typical piglet behaviors, including lying, sitting, standing, drinking, suckling, mounting, head knocking, and biting ear. These behaviors represent key behavioral traits during piglet growth, serving as indicators of their health status and shedding light on their interactions with the environment. These insights provide valuable references for farm management and health evaluation.

The data collection was conducted at the commercial pig farm mentioned in the previous section, utilizing an Intel RealSense depth camera D435i (Intel Corporation, Santa Clara, CA, USA) to record piglet behaviors. As shown in the “Data Collection” section of Figure 1, the camera was mounted in an overhead position at a height of 2–4 m above the ground, ensuring comprehensive coverage of the piglet activity area and effectively minimizing the loss of behavioral information caused by occlusions. The data collection spanned two months, from mid-July to mid-September 2017. To ensure high-quality image data, the camera resolution was set to 1920 × 1080, providing clear visual information for subsequent behavior recognition and quantitative analysis. Throughout the data collection process, the installation angle of the camera was strictly controlled, and adjustments were made according to the pig house’s environmental conditions to minimize the influence of external variables on data quality. These measures ensured the scientific rigor and consistency of the behavioral data.

**Figure 2:**
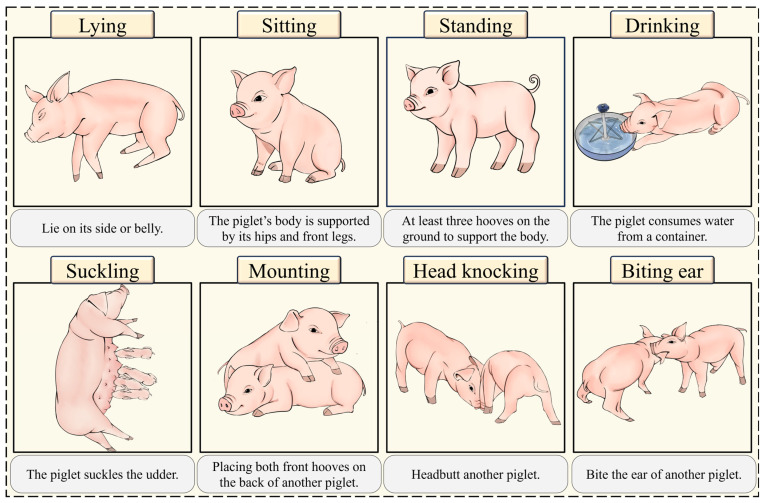
Recognition rules of typical behaviors of piglets.

Additionally, Figure 2 provides detailed definitions of each behavior along with corresponding examples, offering a standardized reference framework for subsequent data annotation and classification.

#### 2.1.3. Data Pre-Processing

In the task of piglet behavior recognition, we implemented rigorous quality control measures to ensure the effectiveness and robustness of model training. The data preprocessing pipeline comprised three key steps: data extraction, annotation, and augmentation. First, PNG-format images were extracted from raw videos using a Python script (version 3.9.0), with a sampling interval of one frame per 15 frames to maintain data continuity and coverage. A total of 1300 images were ultimately selected as the foundation of the dataset, with each image containing multiple instances of piglet behaviors.

For the data annotation process, we utilized the LabelImg software (https://github.com/HumanSignal/labelImg, accessed on 1 January 2025) to annotate piglet behaviors in each image, strictly adhering to the behavior classification criteria defined in Figure 2. Additionally, to ensure the reliability of the annotations, senior researchers with over 10 years of experience in piglet farming were invited to manually review the quality of the labels.

Given the inherent randomness of piglet behaviors and the brief duration of certain actions, class imbalance was observed within the dataset. To mitigate the bias introduced by class imbalance, a series of data augmentation techniques, including horizontal rotation, Vertical rotation, Random brightness, Contrast adjustment, Gaussian noise, Random erasure, stretching transformation, and HSV color space transformation, were applied to increase the number of images for minority classes, thereby achieving balanced class distributions and improving the model’s robustness and generalization performance. Each augmentation method contributed 100 additional images, expanding the dataset to a total of 2100 images. As shown in the “Data Augmentation” section of Figure 1, examples of these data augmentation techniques are displayed.

**Figure 3:**
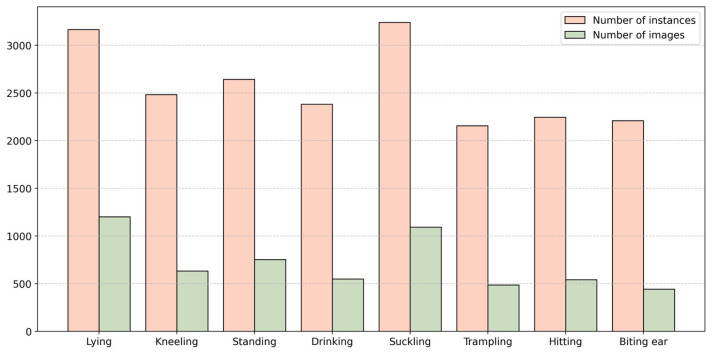
Number of instances and images for each category based on the dataset.

To ensure consistency with the YOLO model’s training requirements without compromising the recognition accuracy, all images were resized to 640 × 640 pixels. The dataset was then divided into training, validation, and test sets in a 7:2:1 ratio, resulting in 1470 images for training, 420 for validation, and 210 for testing. Each image contained multiple instances of piglet behavior, with the entire dataset encompassing 20,529 behavior instances. Figure 3 shows the number of instances and images for each category in the dataset after data augmentation.

### 2.2. Methods

#### 2.2.1. Standard YOLOv8 Model

**Figure 4:**
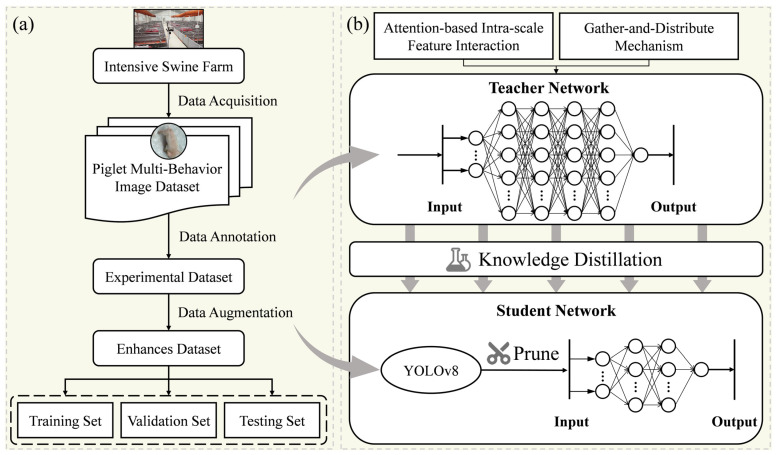
Workflow diagram of this study. (**a**) The piglet multi-behavior recognition dataset is constructed by acquiring images from intensive pig farming environments, followed by annotation and data augmentation; (**b**) the teacher model is built by combining Attention-based Intra-scale Feature Interaction with a multi-scale feature fusion network based on the “Gather-and-Distribute” mechanism. The original YOLOv8 model is pruned to reduce its parameter size, and finally, the teacher model is used for knowledge distillation to train the student model.

The YOLO (You Only Look Once) series models [32,33,34,35,36] have been widely applied in various recognition tasks due to their outstanding performance and efficiency. In this study, we used YOLOv8 as the foundational framework for our model. The whole workflow is shown in Figure 4. The architecture of YOLOv8 primarily consists of three components: the backbone network, the neck network, and the detection head.

The backbone network consists of convolutional modules (Conv), C2f feature enhancement modules, and a fast spatial pyramid pooling (SPPF) module [37]. Specifically, the C2f module enhances feature representation through gradient splitting and residual connections, while the SPPF module effectively aggregates multi-scale contextual information using cascaded max pooling. The neck network is designed as a bidirectional feature aggregation architecture, integrating the bottom-up detail propagation of the Path Aggregation Network (PAN) [38] with the top-down semantic enhancement mechanism of the Feature Pyramid Network (FPN) [39] to optimize cross-level feature interactions. The detection head employs a decoupled design, separating the classification and regression branches to reduce task interference, and utilizes an anchor-free mechanism to directly predict the geometric parameters of the target, significantly simplifying the post-processing workflow. This approach accelerates model convergence and improves the overall detection performance.

#### 2.2.2. The Overall Architecture of the Teacher Model

**Figure 5:**
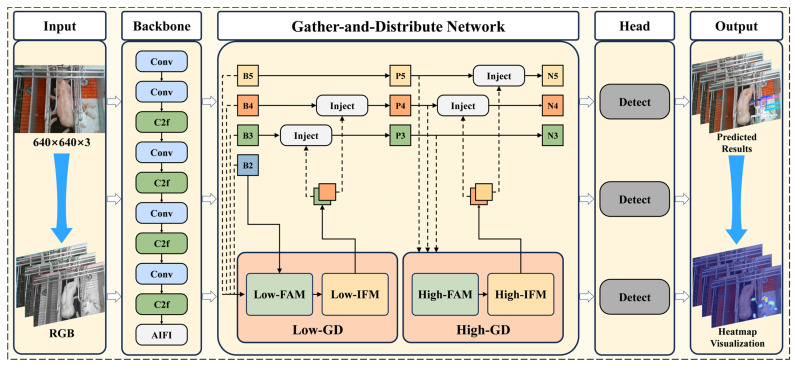
The architecture of the teacher model.

Compared to traditional livestock detection tasks, piglet behavior recognition presents significantly higher complexity. This complexity arises primarily from the following factors: piglets exhibit high activity levels and often display multiple behaviors within a short time frame; furthermore, frequent body occlusions between individuals further complicate the recognition process. Although the YOLOv8 model is capable of handling such tasks, it often encounters limitations in detection accuracy under complex scenarios. Additionally, the large parameter size of YOLOv8 poses challenges for deployment on edge devices, particularly in real-time applications. To achieve high-accuracy piglet behavior recognition while enhancing the model efficiency and suitability for edge deployment, this study introduces several optimizations to the YOLOv8s model architecture. The improved network structure, as shown in Figure 5, includes the following:

- An attention-based intra-scale feature interaction (AIFI) structure is introduced to replace the spatial pyramid pooling-fast (SPPF) layer in the backbone network. This enhancement optimizes feature representation and interaction, bolstering the model’s capacity to process high-level semantic information and improving its ability to handle complex semantic features;
- In the neck network of YOLOv8, a Gather-and-Distribute (GD) mechanism is introduced to facilitate the efficient fusion of multi-scale feature information. This mechanism effectively reduces feature loss between different layers, enhancing the model’s ability to integrate features across multiple scales, which in turn improves the detection accuracy.

#### 2.2.3. Attention-Based Intra-Scale Feature Interaction

**Figure 6:**
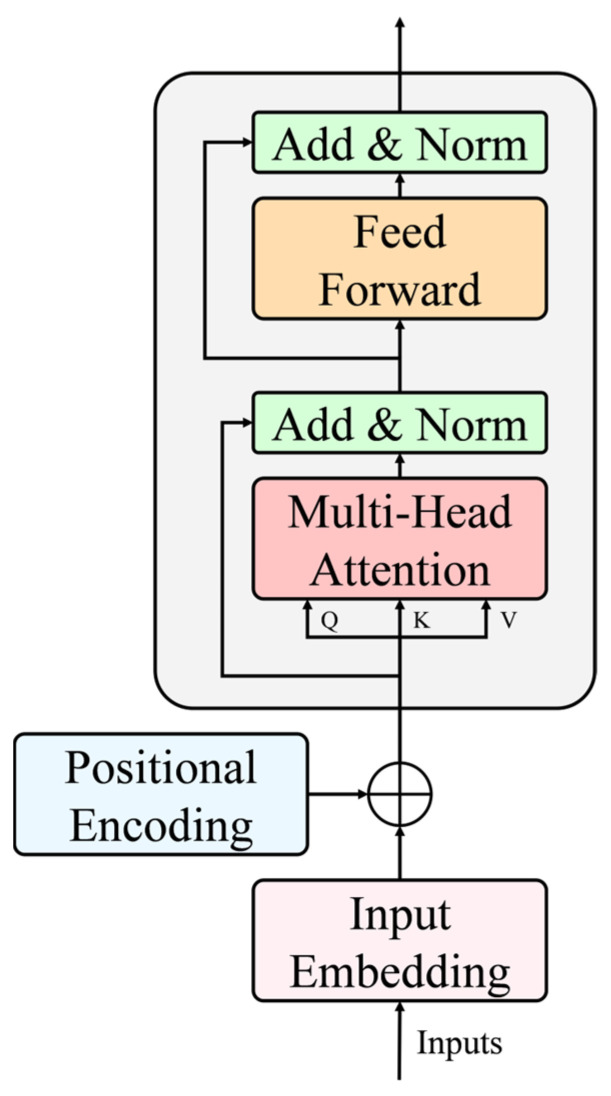
Attention-based Intra-scale Feature Interaction module structure diagram.

In pigpens, piglets are often obstructed by the barriers or the bodies of other piglets, leading to a decrease in the accuracy of behavior-recognition models, and in some cases, resulting in missed detections or false positives. To address this issue, this paper introduces the Attention-based Intra-scale Feature Interaction (AIFI) module [40]. The Transformer-based AIFI module enhances feature representation by capturing long-range dependencies through self-attention, effectively overcoming the locality limitation of traditional convolutions. This capability is particularly beneficial in complex pigpen environments with frequent occlusions, enabling more accurate and robust behavior recognition [41]. The overall structure of the AIFI module is shown in Figure 6.

The AIFI module consists of two core components: Multi-Head Attention and Feed Forward Network (FFN). Initially, AIFI embeds the input 2D image into a 1D vector for further processing. To enable the model to leverage the positional information of the input, we incorporate “positional encodings” into the input embeddings. These encodings are computed using sine and cosine functions of different frequencies:

(1) $$PE(pos,2i)=sin\left(\frac{pos}{10000^{\frac{2i}{dim}}}\right)$$

(2) $$PE(pos,2i+1)=cos\left(\frac{pos}{10000^{\frac{2i}{dim}}}\right)$$

where pos is the position, i is the dimension index, and dim refers to the model’s dimensionality. In other words, each dimension of the positional encoding corresponds to a sinusoid.

Subsequently, the Multi-Head Attention mechanism is applied to perform multiple sets of self-attention calculations, capturing information from different positions and thereby enhancing the model’s ability to handle long-range dependencies.

For the input feature map $F\in R^{H\times W\times C}$, the feature map is reshaped into a 2D matrix $X\in R^{N\times C}$ through a flattening operation, where N=H×W is the number of pixels in the image. The AIFI module then computes the Query vector (*Q*), Key vector (*K*), and Value vector (*V*) by applying linear projections using their respective weight matrices to the reshaped input matrix X. The equations are as follows:

(3) $$Q_{i}=X\cdot W_{i}^{q},\;\;\;\;\;\;K_{i}=X\cdot W_{i}^{k},\;\;\;\;\;\;V_{i}=X\cdot W_{i}^{v}$$

where $W_{i}^{q}$, $W_{i}^{k}$, $W_{i}^{v}$ are the weight matrices corresponding to the Query, Key, and Value vectors, respectively. The index *i* refers to the attention head in the Multi-Head Attention mechanism, where each head has its own independent weight matrices.

The self-attention mechanism is employed to encode contextual information related to global features. It computes the attention values according to the following equation:

(4) $${Attention}_{i}(Q_{i},K_{i},V_{i})=softmax\left(\frac{Q_{i}^{T}\cdot K_{i}}{\sqrt{d_{k}}}\right)\cdot V_{i}$$

where $d_{k}$ denotes the dimensionality of the key vector $K_{i}$, and the softmax function serves as an activation function to normalize the computed dot product results.

Subsequently, in the Multi-Head Self-Attention (*MHSA*) mechanism, multiple independent attention heads are computed in parallel. Their outputs are then concatenated and projected back to the original dimensionality via a linear transformation:

(5) $$MHSA(X)=Concat\left({Attention}_{1},{Attention}_{2},\ldots ,{Attention}_{h}\right)\cdot W^{o}$$

where *h* represents the number of attention heads, and $W^{o}\in R^{C\times C}$ is a learned weight matrix used to project the outputs of the multiple attention heads back to the original dimension.

Afterwards, the sequence undergoes residual connections and normalization before passing through the feed-forward network (FFN). This process introduces non-linearity, enabling the model to learn complex relationships between different elements of the sequence. Finally, the 1D vector, processed by the feed-forward network, is converted back into a 2D form for subsequent operations. The formal definition of the feed-forward network is as follows:

(6) $$FFN(X)=W_{2}\cdot \sigma (W_{1}\cdot I)$$

where W represents the parameter matrices, σ is the GELU activation function, and I is the input after being processed by the self-attention mechanism.

#### 2.2.4. Gather-And-Distribute Mechanism

**Figure 7:**
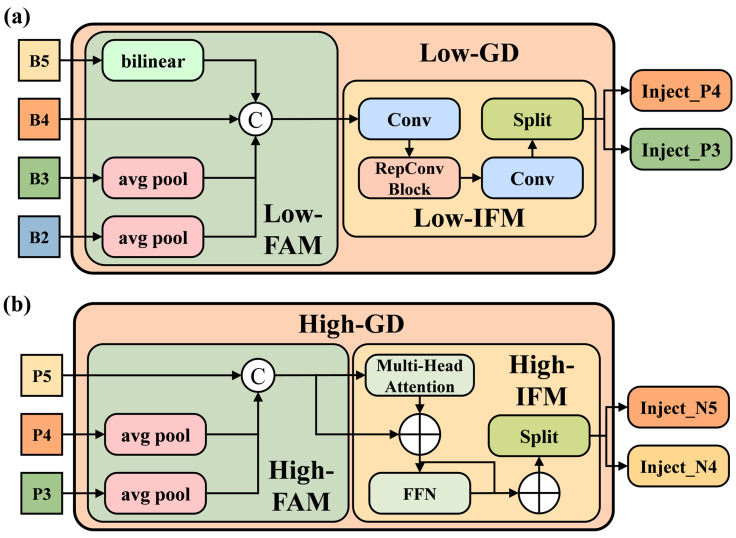
Gather-and-Distribute mechanism. (**a**) Low-GD branch; (**b**) High-GD branch.

In the task of piglet behavior recognition, distinct behavior patterns typically manifest across various spatial scales. However, Wang et al. [42] pointed out that the traditional FPN-PAN network structure adopted by the YOLOv8 algorithm, despite its multi-scale feature fusion capability, exhibits the following limitations: it relies on multiple branches to fuse features from adjacent layers, while non-adjacent layer information can only be accessed indirectly and recursively. This transmission mechanism is prone to information loss, particularly when addressing complex behavior patterns. To overcome this limitation, this study designs a neck network structure based on a Gather-and-Distribute (GD) mechanism [42]. This structure globally fuses features from different layers, strengthens the modeling of the global context, and injects global information into each layer to enhance feature representation. The improved neck network structure consists of two main branches: Low-stage Gather-and-Distribute (Low-GD) and High-stage Gather-and-Distribute (High-GD). Each branch has gathering and distributing processes, including the Feature Alignment Module (FAM), the Information Fusion Module (IFM), and the Information Injection Module (Inject Module). The overall structure of the GD mechanism is shown in Figure 7.

The Low-GD branch aggregates and integrates features [*B*2, *B*3, *B*4, *B*5] from the backbone network to preserve high-resolution features, thereby effectively retaining complex object information and fine-grained details. The structure of Low-GD is illustrated in Figure 7a.

First, the Low-FAM utilizes average pooling layers to downsample the input features [*B*2, *B*3, *B*4, *B*5], standardizing the spatial dimensions of the feature maps and aggregating information from multiple layers. This process generates the aligned features $F_{align}$:

(7) $$F_{align}=Low\_FAM([B2,\;B3,\;B4,\;B5])$$

The Low-IFM employs multi-layer reparametrized convolutional blocks (RepBlock) and then splits into two parts by channel to obtain global feature information [$F_{inj\_P3},F_{inj\_P4}$].

(8) $$F_{global}={[F}_{inj\_P3},F_{inj\_P4}]=Split\left(RepBlock(F_{align})\right)$$

**Figure 8:**
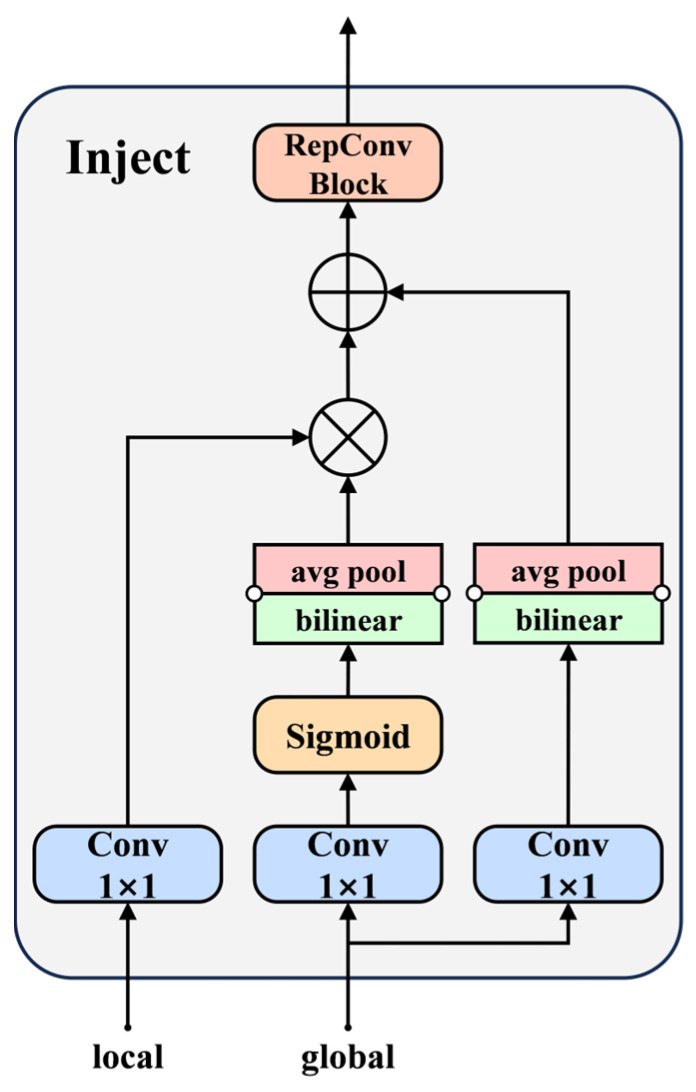
Structural diagram of Inject.

Finally, the Inject module uses attention operations to merge local features $F_{local}$[*B3*, *B4*], representing the current input scale, with global features $F_{global}$[$F_{inj\_P3},F_{inj\_P4}$], generated by the Low-IFM module. These features are fused after being scaled through bilinear interpolation or average pooling. The resulting features are further refined via the RepConv block, with the final outputs represented as *P3*, *P4*, and *P5*. The detailed structure of the Inject module is illustrated in Figure 8.

The High-GD branch further integrates the features [*P*3, *P*4, *P*5] generated by Low-GD to enhance high-level semantic information fusion, as illustrated in Figure 7b. First, the *High-FAM* standardizes the spatial dimensions of the input features using average pooling, generating the aligned feature $F_{align}$, which simplifies subsequent feature aggregation:

(9) $$F_{align}=High\_FAM([P3,P4,P5])$$

Unlike Low-IFM, the High-IFM employs a Transformer block to enhance the global information modeling capability. The Transformer block performs the in-depth fusion of global contextual features, generating the aggregated feature $F_{fuse}$. Subsequently, a 1 × 1 convolution operation and channel splitting are applied to $F_{fuse}$, dividing it into multiple global feature information [$F_{inj\_N4}$,$\;F_{inj\_N5}$]:

(10) $$F_{fuse}=Transformer\left(F_{align}\right)$$

(11) $$F_{global}=F_{inj\_N4},F_{inj\_N5}=Split\left(Conv1\times 1\left(F_{fuse}\right)\right)$$

The Transformer block comprises a multi-head attention block, a feed-forward network (FFN), and residual connections, ensuring efficient global feature extraction and fusion.

The Inject module in High-GD is designed similarly to that in Low-GD, where the fused global features are injected into different feature layers. This process produces semantically enriched high-level fused features [*N*3, *N*4, *N*5], which are subsequently fed into the detection network to enhance the overall detection performance.

#### 2.2.5. Channel Pruning Based on LAMP

**Figure 9:**
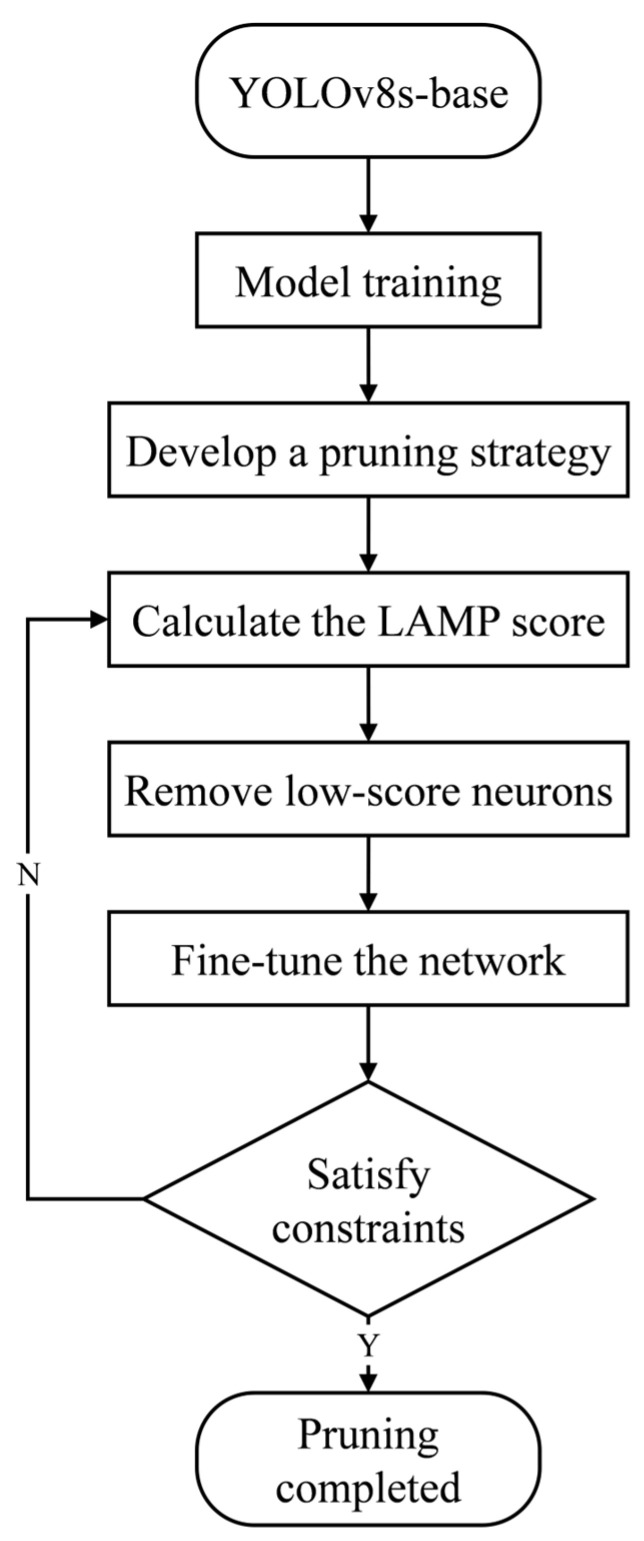
Model pruning flowchart.

Due to the limited computational resources of mobile devices, the model parameter size significantly impacts the ability to perform detection tasks quickly and efficiently. In resource-constrained environments, model pruning techniques can substantially reduce the computational overhead, thereby enhancing the deployment efficiency on edge devices. Therefore, pruning optimization of the trained YOLOv8s model is crucial. To minimize model complexity while maintaining maximum detection accuracy, this study employs a pruning method based on layer-adaptive pruning method (LAMP) [43]. The overall pruning process is illustrated in Figure 9.

LAMP is a global network optimization technique that centers around assigning scores to the weights in each layer, allowing for the removal of connections with scores below a specific LAMP threshold, rather than simply eliminating connections with weights below a fixed threshold in each layer. This layer-adaptive pruning strategy dynamically adjusts the pruning sparsity of each layer based on the importance of the weights, effectively avoiding the performance degradation that arises from the uniform threshold approach in traditional pruning methods. LAMP introduces an innovative weight evaluation and pruning mechanism that is computationally efficient, requiring neither hyperparameter tuning nor relying on the prior knowledge of specific models.

Specifically, the calculation of the LAMP score is based on the relative importance of the weights. For each layer’s weight tensor $W^{\left(l\right)}$, it is first flattened into a one-dimensional vector $w^{\left(l\right)}=[w_{1}^{\left(l\right)},w_{2}^{\left(l\right)},\ldots ,w_{n_{l}}^{\left(l\right)}]$ and then sorted in ascending order of the absolute values of the weights, i.e., $\left|w_{1}^{\left(l\right)}|\leq |w_{2}^{\left(l\right)}|\leq \ldots \leq |w_{n_{l}}^{\left(l\right)}\right|$. The LAMP score for the *u*th weight is then calculated using the following formula:

(12) $${Score}_{u}^{\left(l\right)}=\frac{{\left(w_{u}^{\left(l\right)}\right)}^{2}}{\sum_{v\geq u}\;{\left(w_{v}^{\left(l\right)}\right)}^{2}}$$

where $w_{u}^{\left(l\right)}$ represents the *u*th sorted weight in layer l, and the denominator $\sum_{v\geq u}\;{\left(w_{v}^{\left(l\right)}\right)}^{2}$ denotes the sum of the squared weights starting from index u.

The LAMP ${Score}_{u}^{\left(l\right)}$ reflects the relative importance of this weight in comparison to the remaining weights within the same layer. A higher score indicates that the weight contributes more significantly to the model’s performance. During the pruning process, weights with higher LAMP scores are prioritized for retention, while those with lower scores are removed, thereby achieving a balance between reducing model complexity and preserving performance.

**Figure 10:**
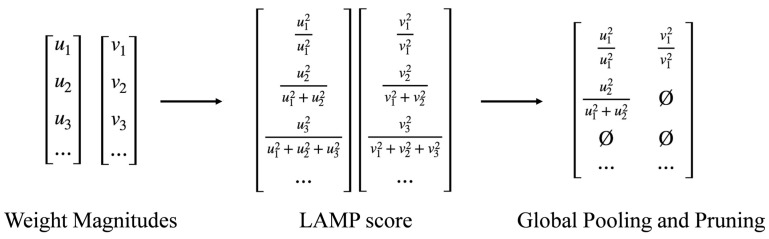
Schematic diagram of the LAMP score calculation process and its application to global pruning.

In the task of piglet behavior recognition, the LAMP method demonstrates significant advantages. Due to the diversity and dynamics of piglet behavior, the model must exhibit high generalization capability and real-time performance. By applying LAMP pruning, we are able to significantly reduce the model’s parameter count and computational overhead while maintaining the detection accuracy, making it more suitable for deployment on resource-constrained edge devices. As shown in Figure 10, the calculation of the LAMP score only requires simple squaring and normalization operations based on the weights, without the need for additional hyperparameters or reliance on the structural information of specific models. This design allows LAMP to be broadly applied to different types of neural network layers (e.g., fully connected layers and convolutional layers), providing an efficient and reliable optimization solution for piglet behavior recognition models.

#### 2.2.6. Knowledge Distillation

Existing methods often use deep networks to extract fine-grained features for accurate piglet behavior recognition in complex farm environments. However, this significantly increases the model complexity and hinders deployment on edge devices. To address the conflict between model accuracy and lightweight requirements, this study introduces knowledge distillation techniques into the field of livestock behavior recognition, proposing an optimization approach for the YOLO-Piglet model based on BCKD.

Knowledge distillation constructs a “teacher-student” framework for knowledge transfer, allowing implicit knowledge from the complex teacher model to be effectively transferred to a lightweight student model. This process enhances the student model’s performance and generalization ability without altering its structure, effectively improving the smaller model’s accuracy while meeting real-time and deployment requirements.

Unlike traditional methods, which solely rely on soft probability distributions for knowledge transfer, BCKD [44] is a novel knowledge distillation method specifically designed for complex object detection tasks, aiming to address the inefficiency of traditional classification distillation in such tasks. In behavior recognition tasks, the imbalance between foreground categories causes traditional knowledge distillation methods to overlook the absolute classification scores for each category. This oversight may lead to an optimized distillation loss function, but it does not necessarily ensure optimal classification performance in the student model. The core idea of BCKD is to transform multi-class classification tasks into multiple binary classification tasks, applying knowledge distillation independently to each binary classification task.

**Figure 11:**
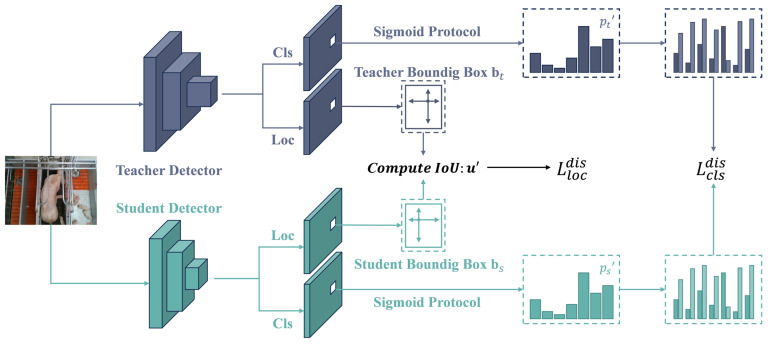
Architecture diagram of piglet multi-behavior detection model based on knowledge distillation.

The distillation process of BCKD is illustrated in Figure 11. This method combines two innovative distillation loss functions specifically designed for object detection tasks:

1. The binary classification distillation loss $L_{cls}^{dis}$, which represents the classification logits as multiple binary mappings, and it extracts classification knowledge through a distillation loss similar to binary cross-entropy;
2. The IoU-based localization distillation loss $L_{loc}^{dis}$, which calculates the Intersection over Union (IoU) between the predicted bounding boxes of the models and applies an IoU loss to transfer localization knowledge from the teacher model to the student model.

During the distillation process, the classification logits are treated as multiple binary classification mappings. The classification scores $p^{t^{\prime }}$ and $p^{s^{\prime }}$ are obtained by calculating $p^{t^{\prime }}=Prot_{Sig}(l^{t})$ and $p^{s^{\prime }}=Prot_{Sig}(l^{s})$, respectively. Subsequently, the classification distillation loss is computed based on these binary classification scores:

(13) $$L_{BCE}(p_{i,j}^{s}\prime ,p_{i,j}^{t}\prime )=-\left(\left(1-p_{i,j}^{t}\prime )\cdot log(1-p_{i,j}^{s}\prime )+p_{i,j}^{t}\prime \cdot log(p_{i,j}^{s}\prime \right)\right)$$

(14) $$L_{cls}^{dis}(x)=\sum_{i=1}^{n}\;\sum_{j=1}^{K}\;{L_{BCE}(p_{i,j}^{s}}^{\prime },{p_{i,j}^{t}}^{\prime })$$

where $L_{cls}^{dis}$ represents the classification distillation loss, $L_{BCE}$ denotes the binary cross-entropy loss, and ${p_{i,j}^{s}}^{\prime }$ and ${p_{i,j}^{t}}^{\prime }$ correspond to the *i*th position and the *j*th class of $p^{s^{\prime }}$ and $p^{t^{\prime }}$, respectively.

A loss weighting strategy is also employed to focus on extracting important samples. The importance weight w of sample x is computed as follows:

(15) $$w=\left|p^{t^{\prime }}-p^{s^{\prime }}\right|$$

where $w\subseteq R^{n\times K}$. Each element in *w* is used to weight the classification distillation loss of sample *x*.

Therefore, the final classification distillation loss is expressed as follows:

(16) $$L_{cls}^{dis}(x)=\sum_{i=1}^{n}\;\sum_{j=1}^{K}\;w_{i,j}\cdot {L_{BCE}(p_{i,j}^{s}}^{\prime },{p_{i,j}^{t}}^{\prime })$$

Localization is a crucial aspect of object detection. The unstructured IoU localization distillation loss is employed, using the Intersection over Union (IoU) between two bounding boxes as the distillation objective, thereby transferring localization knowledge directly from the teacher model to the student model. This method involves obtaining the detection boxes from both the teacher and student models, with the predictions for the *ith* position of input sample x represented as $o_{i}^{t}$ and $o_{i}^{s}$ for the teacher and student models, respectively. The bounding boxes are obtained using anchor positions $A_{i}$ and the predicted results via $b_{i}^{t}=Decoder(A_{i},o_{i}^{t})$ and $b_{i}^{s}=Decoder(A_{i},o_{i}^{s})$. The IoU between $b_{i}^{t}$ and $b_{i}^{s}$ is denoted as $u_{i}^{\prime }$. The local distillation loss is then calculated as follows:

(17) $$L_{loc}^{dis}(x)=\sum_{i=1}^{n}\;max(w_{j})\cdot (1-u_{i}^{\prime })$$

where $max(w_{j})$ represents the maximum weight within the class, emphasizing the critical localization information.

The total distillation loss function combines binary classification distillation loss and IoU-driven localization distillation loss using a weighted fusion strategy. This design achieves synchronized improvement in classification feature semantic alignment and bounding box regression accuracy through a dual-task collaborative optimization mechanism. Its computation is expressed as follows:

(18) $$L_{total}^{dis}(x)=\alpha_{1}\cdot L_{cls}^{dis}(x)+\alpha_{2}\cdot L_{loc}^{dis}(x)$$

where $\alpha_{1}$ and $\alpha_{2}$ are two hyperparameters representing the loss weights for the classification distillation loss and the localization distillation loss, respectively.

These carefully engineered loss functions comprehensively address the inherent foreground–background class imbalance in object detection tasks, while simultaneously accounting for the critical impact of localization precision on model performance. Through these meticulously engineered loss functions, we achieve substantially improved training efficacy and optimization of the student model while maintaining architectural compactness.

## 3. Results and Analysis

### 3.1. Experiment Environment

The computational infrastructure for this study comprised an NVIDIA GeForce RTX 3080 Ti GPU (Nvidia, Santa Clara, CA, USA) with 12 GB VRAM, coupled with an Intel Core i7-10700 processor (2.9 GHz base clock, 16-core architecture (Intel, Santa Clara, CA, USA)) and 32 GB DDR4 RAM. To ensure reproducibility, all experiments were conducted on a dedicated computation platform running Ubuntu 22.04.4, with Python package management carried out through Anaconda 23.1.0.

The deep learning framework was implemented in Python 3.8 using PyTorch 1.11.0, with CUDA 11.3 and cuDNN 8.2.0 acceleration libraries for GPU-accelerated computations. All experiments in this study maintained identical initialization seeds and parallel processing configurations, with the critical hyperparameter settings detailed in Table 1.

### 3.2. Evaluation Indicators

This study establishes a multi-dimensional evaluation framework to comprehensively assess algorithm performance through two principal dimensions: model accuracy and computational efficiency. The model accuracy is characterized by the precision (*P*), recall (*R*), and mean average precision (*mAP*), while computational efficiency is evaluated through the parameter count (Params), weights (Model Size), floating-point operations per second (FLOPs), and single-frame inference time. The mathematical formulations of these metrics are defined as follows:

(19) $$P=\frac{TP}{TP+FP}\times 100\%$$

(20) $$R=\frac{TP}{TP+FN}\times 100\%$$

(21) $$AP=\int_{0}^{1}\;P\left(R\right)dR$$

(22) $$mAP=\frac{1}{k}\sum_{i=1}^{k}AP_{i}$$

Among them, true positives (*TP*) represent the number of piglet behaviors correctly detected by the model; false positives (*FP*) denote the number of behaviors wrongly detected by the model as other behaviors; false negatives (*FN*) indicate the number of piglet behaviors that the model fails to detect. Precision is the ratio of *TP* to the sum of *TP* and *FP*, which is used to measure the correctness of the model’s detection results for the recognized behaviors. Recall is the ratio of *TP* to the sum of *TP* and *FN*, evaluating the model’s ability to capture positive samples. The average precision (*AP*) is the area under the precision—recall (*P*—*R*) curve, and it is the average *AP* of *k* (*k* = 8) categories of group-living piglet behaviors. The number of parameters refers to the quantity of parameters within the model, which indicates the size of the model. Smaller models are easier to deploy in various application scenarios. On the other hand, FLOPs quantify the computational complexity of the algorithm. Models with lower FLOPs are considered to have lower requirements in terms of hardware conditions. The single-frame inference time is the time required for a single input sample to undergo forward propagation and obtain the output results, reflecting the model’s performance in terms of resource consumption and the response time.

### 3.3. Experiments on Model Pruning

Different pruning methods and pruning rates significantly affect the accuracy of the pruned model for the same model, where the pruning rate represents the compression ratio of computational costs. To investigate the impact of various pruning methods and pruning rates on model performance, this section conducts pruning experiments on the piglet multi-behavior dataset. By comparing the performance metrics of the YOLOv8s model under different pruning methods and rates, this section aims to assess the impact of pruning on the model and provide a basis for selecting the optimal student model.

#### 3.3.1. Comparison of Different Pruning Methods

To evaluate the effectiveness of different pruning methods, this study conducted experiments on five commonly used pruning techniques: LAMP, Slim [45], Group_slim [46], Group_hessian [47], and Group_taylor [48]. In this experiment, a pruning rate of 1.5 was applied, and global channel pruning was employed, ensuring that the number of pruned channels in each layer was approximately consistent. YOLOv8s was used as the baseline model, and a comparison was made with the five pruning methods. The experimental results are shown in Table 2.

The experimental results show that among the five pruning methods, the LAMP method achieves the best overall performance. Compared to the baseline model, the LAMP method improved accuracy by 0.3% and recall by 3.4%. At the same time, FLOPs were reduced by approximately 33.7%, and both the number of parameters and model weights decreased by about 58%. These results demonstrate that the LAMP method effectively enhances model performance while significantly reducing the computational complexity and storage requirements.

#### 3.3.2. Comparison of Different Pruning Rates

To investigate the impact of the pruning rate on model performance, this experiment selected LAMP as the pruning method and set four different pruning rate gradients: 1.5, 2.0, 2.5, and 3.0. A pruning rate of 2.0 means that the floating-point operations (FLOPs) of the pruned model are half of the original model, with 50% of the channel connections in the original network being removed. After pruning, the network requires fine-tuning to compensate for the lost connections, restore accuracy, and improve the overall network performance. Detailed information for each group is provided in Table 3.

The experimental results indicate that as the pruning rate increases, the number of model parameters and computational complexity significantly decrease. When the pruning rate is set to 2.0, the model achieves its best performance, with an accuracy of 86.8%, a recall of 84.1%, and an mAP of 87.8%. This result suggests that a pruning rate of 2.0 strikes the optimal balance between performance and computational efficiency. Although further increases in the pruning rate (e.g., 2.5 and 3.0) reduce the computational complexity and parameter count even more, the model performance drops significantly. Particularly, when the pruning rate is 3.0, accuracy and recall decrease to 53.7% and 52.1%, respectively, demonstrating that excessive pruning negatively impacts the model performance.

**Figure 12:**
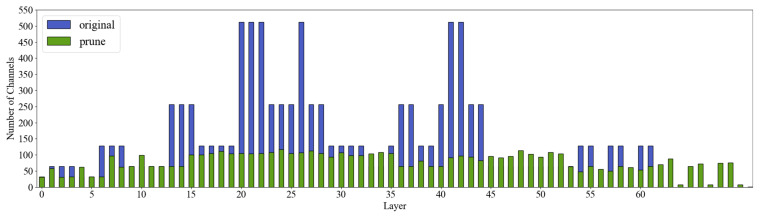
Comparison of the number of channels before and after pruning.

Ultimately, we selected the LAMP pruning method and set the pruning rate to 2.0 to retain more important connections in the shallow feature extraction layers, while applying more aggressive pruning to the redundant layers. This approach significantly reduces the computational complexity and the number of parameters in the model while maintaining its performance. To further understand the changes in the channels during the pruning process, we present the channel diagram of the pruned model. Figure 12 compares the pruning channels before and after pruning for each layer, where the horizontal axis represents the names of the layers in the network and the vertical axis represents the number of parameters for each layer. This comparison allows us to visually observe the impact of pruning on the network structure at different levels and how pruning effectively optimizes the parameter configuration of the network.

### 3.4. Experiments on the Improved Model

#### 3.4.1. Comparison of Different Neck Networks

In order to evaluate the effectiveness and advantages of the Gather-and-Distribute (GD) mechanism for piglet multi-behavior recognition in the complex environment of pig farms, we conducted systematic experiments under consistent training conditions. This section compares the GD mechanism with several mainstream Neck Networks, including Bidirectional Feature Pyramid Network (BiFPN) [49], Hybrid-Scale Feature Pyramid Network (HS_FPN) [50], and Global Feature Pyramid Network (GFPN) [51]. In the experiments, we replaced the original FPN-PAN network of YOLOv8 with the aforementioned Neck Networks to assess the performance differences among these methods in practical applications. The detailed experimental results are presented in Table 4.

The experimental results show that, compared to other Neck Networks, the GD mechanism achieved the highest performance in piglet multi-behavior recognition. Its precision, recall, and mAP all surpassed those of other Neck Networks. Specifically, the precision of the GD mechanism reached 87.9%, the recall was 85.2%, and the mAP was 87.8%. Compared to FPN-PAN, BiFPN, HS_FPN, and GFPN, the precision of the GD mechanism was improved by 1.6%, 4.6%, 2.3%, and 4.2%, respectively.

In the task of piglet multi-behavior recognition, the GD mechanism significantly improved model accuracy compared to other Neck Networks. However, this improvement came with an increase in the computational complexity, parameter count, and model storage requirements. To address these challenges, we applied knowledge distillation to the improved model, allowing the student model to learn the rich feature representations and logical reasoning capabilities of the improved model. This approach enables the student model to reduce the parameter size and computational complexity while maintaining or even surpassing the performance of the teacher model.

#### 3.4.2. Ablation Experiments on the Improved Model

In this section, we use YOLOv8s as the baseline model and optimize it by incorporating the Gather-and-Distribute (GD) mechanism and the AIFI module. To assess the effectiveness of the improvements made to the teacher model, we conducted an ablation study using the controlled variable method. Multiple groups were tested, with the precision (*P*), recall (*R*), mean average precision (mAP@0.5), parameter count, floating point operations, and model size as evaluation metrics.

Experiment A involves replacing the neck network of YOLOv8s with a feature fusion network based on the “Gather-and-Distribute” mechanism for multi-scale feature fusion. Experiment B replaces the SPPF module in the backbone of the original YOLOv8s with the AIFI module. Experiment A + B builds upon Experiment A by additionally replacing the SPPF module in the backbone with the AIFI module. All ablation experiments were conducted under the same dataset and training parameters, with the results shown in Table 5.

The results of the ablation experiments demonstrate that the performance of the model significantly improved with the gradual integration of each modification module. As shown in the table, the baseline model achieved precision, recall, and mAP values of 86.3%, 80.0%, and 87.5%, respectively. When the GD mechanism was introduced (Experiment A), the precision, recall, and mAP increased by 1.6%, 5.2%, and 0.3%, respectively. With the addition of the AIFI module (Experiment B), the precision and recall increased by 1.8% and 6.9%, and the mAP improved by 0.8%. Finally, the combination of both the GD mechanism and AIFI module (Experiment A + B) further enhanced model performance, with precision reaching 88.5%, recall at 87.6%, and mAP at 89.6%. Compared to the baseline model, the precision increased by 2.2%, recall by 7.6%, and mAP by 2.1%, indicating that the design and construction of the network are rational and that the modules are well-compatible.

Furthermore, the results of the ablation experiment also reveal that the GD mechanism in the neck optimization enhances feature fusion, enabling the model to more comprehensively recognize target behaviors. The Attention-based Intra-scale Feature Interaction (AIFI) module better captures the relationships between different conceptual entities within the image, reducing the impact of the occlusions caused by obstacles or other piglets’ bodies. The experimental results validate the optimization and enhancement provided by these two modules in the YOLOv8 backbone and neck, significantly boosting the model’s recognition capabilities.

### 3.5. Experiments on Knowledge Distillation

#### 3.5.1. Comparison of Different Distillation Methods

In this section, we optimize the pruned student model (YOLOv8-prune) using knowledge distillation, with the teacher model being the improved YOLOv8s model (YOLOv8-GDA). The core idea of knowledge distillation is to transfer the knowledge embedded in the complex teacher model to the relatively simpler student model, thereby enhancing the performance of the student model. Knowledge distillation is typically divided into feature distillation and logical distillation, with different distillation methods having a significant impact on the final performance. To investigate the effects of different distillation methods on model performance, this study employs several distillation techniques, including Mean Gradient Distillation (MGD) [52], Correlation Weight Distillation (CWD) [53], L1 [54], L2 [55], and BCKD. We conducted experiments using these distillation methods to evaluate their impact on improving the accuracy and computational efficiency of the student model and selected the most suitable distillation method through a comparative analysis. The detailed results of the experiment are shown in Table 6.

The experimental results indicate that the overall performance of feature distillation methods is suboptimal, with MGD showing the poorest performance, achieving only 88.4% accuracy. In contrast, among the logical distillation methods, BCKD achieved the best results, with an accuracy of 92.6%, recall of 91.2%, and mAP of 91.8%. This suggests that BCKD effectively preserves the generalization ability of the teacher model, significantly enhancing the performance of the student model and demonstrating outstanding results.

#### 3.5.2. Ablation Experiments

We conducted a systematic ablation study on the proposed YOLOv8-Piglet model to evaluate the impact of two optimization techniques, pruning and knowledge distillation, on its performance. The experimental results are summarized in Table 7. The experiment confirms the effectiveness of pruning and distillation in striking a balance between efficiency and accuracy. After undergoing LAMP pruning, the YOLOv8-Piglet model maintained nearly lossless accuracy while significantly reducing the computational demands: parameters were reduced by 74.7%, FLOPs decreased by 50.5%, inference time shortened by 66.5%, and model size shrank by 73.8%. Subsequently, knowledge distillation preserved the same level of model complexity but substantially enhanced the detection performance, increasing the precision by 5.8%, recall by 7.1%, and raising the mAP@0.5 to 91.8%. These results show that pruning improves model efficiency, while knowledge distillation compensates for the loss in representation by transferring semantic priors from the teacher model. Consequently, the student network’s ability to accurately recognize multiple piglet behaviors (suckling, mounting, head knocking, and biting ear) in dynamic and complex scenarios is significantly improved. This approach mitigates the performance degradation of lightweight models under occlusion and lighting changes, while providing a reliable solution for intelligent monitoring in intensive pig farming.

### 3.6. Comparative Experiments of Different Models

To evaluate the effectiveness of the YOLOv8s-Piglet model proposed in this study, we compared it with YOLOv5s, YOLOv7-x, YOLOv8s, and YOLOv10s. Additionally, the performance of the single-stage model SSD, the two-stage model Faster R-CNN, and other object detection models RT-DETR, was also assessed. As shown in Table 8, the experimental results indicate that the FLOPs and parameter counts of SSD, Faster R-CNN, and RT-DETR models are significantly higher than those of other models, resulting in larger model weights. In comparison, the model size of YOLOv8s-Piglet is only 5.9 MB, which is 7.5% of SSD, 6.3% of Faster R-CNN, and 6.9% of RT-DETR. These results suggest that SSD, Faster R-CNN, and RT-DETR do not meet the lightweight real-time detection requirements of piglet behavior, whereas YOLOv8s-Piglet provides an efficient performance with a smaller model size and faster inference speed.

YOLOv8s-Piglet exhibits exceptional accuracy in piglet behavior detection tasks, achieving an accuracy of 92.6%, a recall of 91.2%, and an mAP@0.5 of 91.8%. Compared with other YOLO models—including YOLOv5s, YOLOv7-x, YOLOv8s, and YOLOv10s—YOLOv8s-Piglet demonstrates significant advantages in detection accuracy, parameter efficiency, and model size. Specifically, in terms of the detection accuracy, it outperforms YOLOv5s, YOLOv7-x, YOLOv8s, and YOLOv10s by 19.4%, 20.8%, 6.3%, and 13.3%, respectively; its recall is improved by 13.2%, 16.0%, 11.2%, and 22.1%, respectively—a high recall rate indicates that the model minimizes missed detections, thereby detecting more targets; and for mAP, the advantages are 15.3%, 16.7%, 4.3%, and 17.4%, respectively. The high average precision not only reflects the model’s superior accuracy but also indicates its excellent performance in reducing false positives and adapting to various target behaviors, backgrounds, and environmental conditions.

Furthermore, the experimental results confirm that the YOLOv8s-Piglet model designed in this study exhibits outstanding performance. By employing knowledge distillation, the deep feature representations and contextual reasoning capabilities inherently contained within the teacher model (YOLOv8-GDA) are effectively transferred to the structurally pruned student model (YOLOv8-prune). In this process, the student model not only inherits the teacher model’s precise target-recognition abilities but also significantly reduces the network’s computational complexity and parameter scale, thereby achieving lower inference latency and a smaller model size while maintaining high detection accuracy. This strategy provides a feasible solution for deploying efficient and accurate object detection models in resource-constrained environments.

### 3.7. Deployment Experiments on Edge Computing Devices

In response to the real-time monitoring demands of large-scale farming scenarios, this study proposes a lightweight piglet multi-behavior recognition model architecture for widespread deployment across various farms. To evaluate the practical performance of the proposed YOLOv8-Piglet model on edge computing devices, we selected the NVIDIA Jetson Orin NX as the deployment platform and employed the TensorRT inference library for accelerated optimization. TensorRT is a high-performance inference optimization framework that utilizes techniques such as layer fusion, precision calibration, and automatic kernel tuning to maximize hardware utilization on NVIDIA GPU architectures, thereby significantly enhancing the model’s inference speed. The experimental results are presented in Table 9.

**Figure 13:**
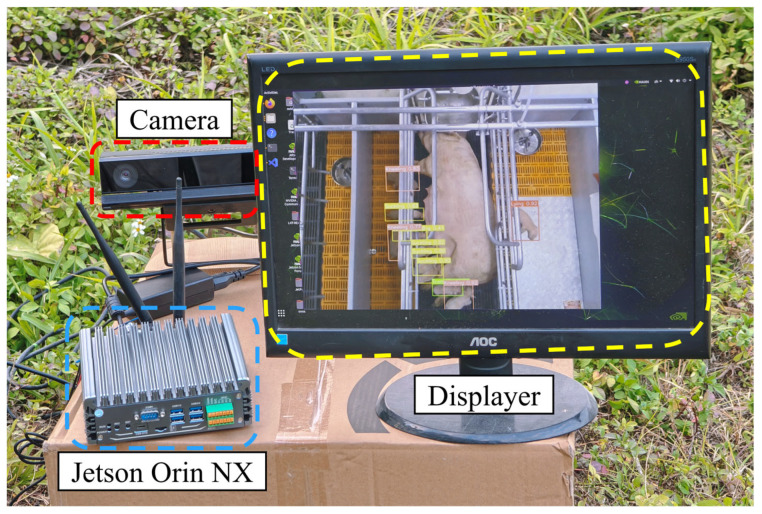
Deployment scenario of the YOLOv8-Piglet model on the Jetson Orin NX development board.

As shown in Table 9, YOLOv8-Piglet demonstrates a significant advantage in inference efficiency across heterogeneous computing environments (Desktop and Jetson Orin NX). On a standardized test set containing 120 images, YOLOv8-Piglet achieves an average single-frame inference time of just 6.6 ms on the desktop, a 66.5% reduction compared to the baseline model YOLOv8s (19.7 ms). On the Jetson Orin NX, YOLOv8-Piglet requires only 163.2 ms, representing a 53.8% decrease from YOLOv8s (353.9 ms). These findings indicate that, through pruning and distillation strategies, YOLOv8-Piglet not only achieves a lightweight model but also significantly enhances the edge-side computational efficiency. Consequently, it meets the stringent low-latency requirements of real-time monitoring systems and is well-suited for deployment on resource-constrained edge computing devices, making it highly promising for widespread adoption in farm environments. The diagram of the actual model deployment is shown in Figure 13.

## 4. Discussion

### 4.1. Comparative Analysis of Piglet Multi-Behavior Recognition Models

**Figure 14:**
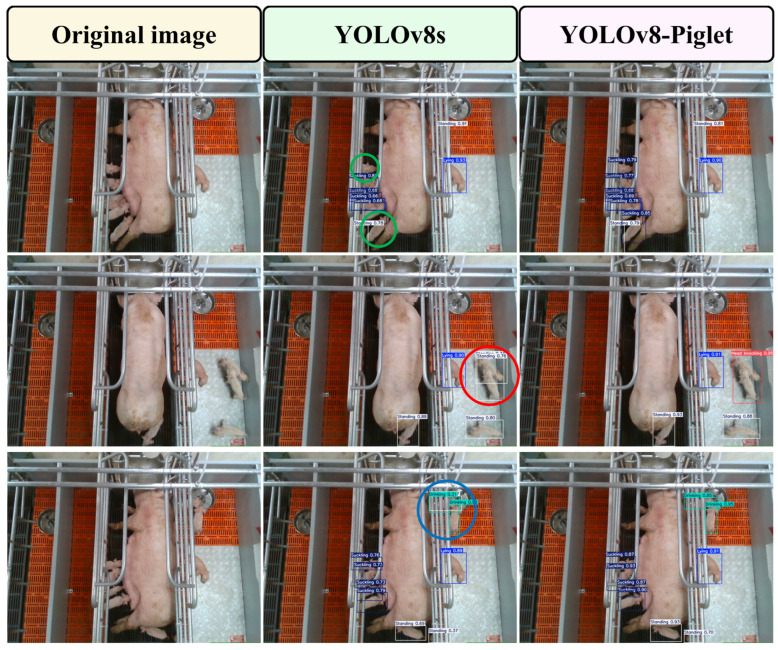
Visualization of the piglet multi-behavior recognition detection results. Green circular markers represent the missed detection samples; red circular markers indicate false positive instances; blue circular markers denote redundant detection results.

To address the challenges posed by mutual occlusion among piglets and partial occlusion by obstacles in large-scale pig farming environments, we conducted empirical validation through the random sampling of images from complex scenarios. This approach was adopted to rigorously evaluate the enhanced YOLOv8-Piglet model’s detection efficacy and environmental adaptability under challenging conditions. Subsequently, a comparative analysis was carried out between the improved YOLOv8-Piglet model and the original YOLOv8s model. The recognition results are presented in Figure 14. The second column of the figure displays the object detection images of the original YOLOv8s model, while the third column presents the object detection images of the YOLOv8-Piglet model after knowledge distillation. As shown in Figure 14, the original YOLOv8s model experiences missed detections and false detections, particularly in complex environments. Specifically, in the 2th image of the 1th row, the original model demonstrates two instances of missed detections. In the region where piglets are positioned close to each other and partially occluded by railings, the model fails to identify one of the piglets. This is likely due to information loss caused by the occlusion, as well as the difficulty in distinguishing piglets in a densely packed environment, which prevents the detection algorithm from accurately recognizing this piglet. Similarly, a piglet near the sow’s hind leg at the bottom of the image is also not detected by the model. This is because the piglet shares a high degree of similarity in shape and color with the sow’s hind leg, making it challenging for the model to effectively differentiate between the two. In the 2th image of the 2th row, a misclassification occurred in the original model. Due to the high visual similarity between complex social interaction behaviors (mounting, head knocking, and biting ear) and simpler actions, the original model erroneously classified the head knocking behavior of piglets as standing behavior. This issue arises from the limitations in the original model’s feature extraction and analysis capabilities, where complex, high-level social interactions are often misinterpreted as simpler, low-level behaviors. This highlights the shortcomings of the original model in recognizing complex social interaction behaviors, making it difficult to accurately distinguish intricate behavior patterns in specific contexts. In the 2nd image of the 3rd row, re-identification issues arise in the original model. During the feature extraction and analysis process, the original model fails to effectively differentiate between subtle variations in similar behaviors. For instance, there is a case where the same pig’s behavior is misclassified as both “drinking” and “standing” simultaneously. When handling behavior recognition tasks in complex and crowded environments, the original model exhibits performance limitations, making it challenging to accurately recognize and distinguish between different behavior patterns with similar visual features.

In contrast, the improved model proposed in this paper enhances the object detection accuracy through knowledge distillation. It not only accurately identifies the high-level social interaction behaviors of piglets, such as mounting, head knocking, and biting ear, but also effectively addresses the occlusion issue, enabling the precise recognition of behaviors involving occluded piglets. This highlights the model’s exceptional adaptability and robustness.

### 4.2. Heat Map Visualization Analysis

**Figure 15:**
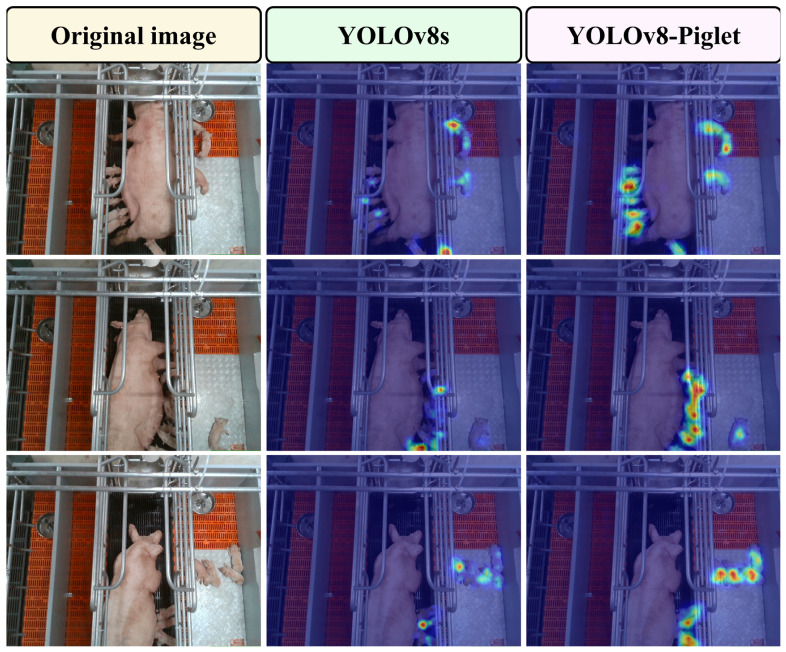
Comparison of heat maps before and after model optimization.

To further assess the effectiveness of our model in recognizing various pig behaviors, we employed the Gradient-weighted Class Activation Mapping (Grad-CAM) [56] method for an interpretability analysis of the model-improvement strategies. Figure 15 presents the heatmaps generated by Grad-CAM on the self-constructed dataset, which provides deeper insights into the multi-dimensional impacts and underlying mechanisms of knowledge distillation on the student model. Following distillation, the YOLOv8-Piglet model demonstrates a significantly improved focus on the target regions. Compared to the original model, it shows a reduction in attention to irrelevant background and non-target areas. In contrast, the original YOLOv8 model struggles to effectively focus on piglet behaviors, resulting in a limited ability to capture these behaviors.

## 5. Conclusions

This study presents a lightweight behavior detection model based on the YOLOv8s architecture, capable of the real-time recognition of eight core piglet behaviors (lying, sitting, standing, drinking, suckling, mounting, head knocking, biting ear). The model accurately tracks individual development trajectories and evaluates group health metrics. By incorporating the Attention-based Intra-scale Feature Interaction (AIFI), the Gather-and-Distribute (GD) mechanism, the Layer-Adaptive Magnitude-Based Pruning (LAMP), and knowledge distillation techniques, the model achieves computational efficiency optimization while maintaining high detection accuracy.

To reduce the impact of occlusion caused by barriers or other piglets’ bodies, we introduced the AIFI module at the end of the backbone network, enhancing the model’s ability to extract features related to piglet behavior. This improvement allows the model to more accurately identify fine-grained behaviors. Additionally, the Gather-and-Distribute (GD) mechanism was incorporated into the neck network to enable lossless cross-layer transmission of feature information. Finally, the improved YOLOv8-GDA model, used as the teacher model, achieved a precision of 88.5%, recall of 87.6%, and mAP of 89.6%. To ensure that the model can adapt to most devices while maintaining detection performance in resource-constrained environments, this study conducted comparative experiments on five different pruning methods. The LAMP method was ultimately selected for pruning, with a pruning rate set to 2.0. Compared to the original model, the YOLOv8-prune model maintained nearly lossless accuracy while reducing the number of parameters by 74.7%, floating point operations (FLOPs) by 50.5%, inference time by 66.5%, and model size by 73.8%. As part of the overall optimization strategy, we applied the BCKD feature knowledge distillation technique, which significantly enhanced the performance of YOLOv8-Piglet without increasing the model complexity. As show in the experimental results, YOLOv8-Piglet outperforms the baseline model in all key metrics. Specifically, compared to the baseline model, YOLOv8-Piglet achieved a 6.3% increase in precision, a 11.2% increase in recall, and an mAP@0.5 of 91.8%, underscoring its superiority in piglet behavior recognition. When deployed on the NVIDIA Jetson Orin NX edge computing platform, the model demonstrates efficient inference speed and exceptional real-time performance. Furthermore, the model is highly scalable and can be applied to other domains, such as individual behavior detection in cattle or behavioral monitoring of small livestock like poultry.

However, there are some limitations to this study. The complex deformations occurring during pig movement—such as limb extension, torso twisting, and skin folding—induce nonlinear rigid-body transformations in the target’s appearance, which significantly increases the ambiguity in feature representation. Existing convolutional neural networks (CNNs) exhibit insufficient feature space representation when modeling such complex deformations. Future work will focus on addressing these challenges, such as by exploring deformation compensation algorithms based on biomechanical models, and incorporating 3D point cloud data augmentation to enhance the model’s ability to generalize across nonlinear deformations.

Furthermore, our research will be extended to explore the extraction of piglet behavior features in more complex environments, with the goal of enhancing the model’s robustness. This approach will enable a more comprehensive understanding of piglet growth patterns and behavioral characteristics, thereby more effectively ensuring their health and welfare, improving farm operational efficiency, and ultimately providing the livestock industry with more reliable, practical, and efficient solutions.

## Figures and Tables

**Table 1:** The training parameters for the experiments.

Hyperparameters	Value
Training epoch	200
Batchsize	8
Optimization	SGD
Learning rate	0.01
Intersection over Union	0.7
Workers	4
Weight decay	0.005
Momentum	0.937
Warm-up momentum	0.8

**Table 2:** Comparison of results of different pruning methods.

**Pruning Method**	***P*** **/** **%**	***R*** **/** **%**	**mAP@0.5** **/** **%**	**FLOPs** **/** **G**	**Param** **/** **M**	**Weights** **/MB**
Base	86.3	80.0	87.5	28.5	11.13	22.5
LAMP	86.6	83.4	86.1	18.9	4.57	9.4
Slim	82.2	78.8	84.2	19.1	6.31	12.9
Group_slim	82.8	79.9	84.6	19.1	8.13	16.7
Group_hessian	80.9	79.5	85.8	19.0	5.25	10.8
Group_taylor	83.7	80.2	82.4	19.0	4.91	10.1

**Table 3:** Comparison of results for different pruning rates.

**Pruning Rate**	***P*** **/%**	***R*** **/%**	**mAP@0.5/%**	**FLOPs/G**	**Param/M**	**Weights** **/MB**
Base	86.3	80.0	87.5	28.5	11.13	22.5
1.5	86.6	83.4	86.1	18.9	4.57	9.4
2.0	86.8	84.1	87.8	14.1	2.82	5.9
2.5	79.1	74.6	81.8	11.3	1.94	4.1
3.0	53.7	52.1	54.3	9.3	1.44	3.1

**Table 4:** Comparison of performance across different Neck Networks.

Neck Network	*P*/%	*R*/%	mAP@0.5/%	FLOPs/G	Param/M	Weights/MB
FPN-PAN	86.3	80.0	87.5	28.5	11.12868	22.5
BiFPN	83.3	71.2	78.3	25	7.367412	15
HS_FPN	85.6	72.1	80.1	23.9	7.136744	14.5
GFPN	83.7	73.6	79.6	29.3	12.12132	24.8
GD	87.9	85.2	87.8	29.9	13.608552	27.7

**Table 5:** Model ablation test with different improvements.

Models	Method		Metrics					
	GD	AIFI	*P*/%	*R*/%	mAP@0.5/%	FLOPs/G	Param/M	Weights/MB
baseline	-	-	86.3	80.0	87.5	28.5	11.13	22.5
A	✓	-	87.9	85.2	87.8	29.9	13.61	27.7
B	-	✓	88.1	86.9	88.3	28.8	12.58	25.4
A+B	✓	✓	88.5	87.6	89.6	30.2	15.06	30.6

Note: ‘✓’ indicates the improvement of the corresponding module.

**Table 6:** Comparison of results of different knowledge distillation methods.

Type	Method	*P*/%	*R*/%	mAP@0.5/%	FLOPs/G	Param/M	Weights/MB
Feature Distillation	MGD	88.4	89.3	88.9	14.1	2.82	5.9
	CWD	89.6	90.1	89.8			
Logical Distillation	L1	91.1	90.3	90.7			
	L2	89.8	90.8	90.5			
	BCKD	92.6	91.2	91.8			

**Table 7:** Results of the ablation experiments on the YOLOv8-Piglet model.

Models	*P*/%	*R*/%	mAP@0.5/%	FLOPs/G	Param/M	Weights/MB
YOLOv8s	86.3	80.0	87.5	28.5	11.13	22.5
+Prune	86.8	84.1	87.8	14.1	2.82	5.9
+Prune + Distill	92.6	91.2	91.8	14.1	2.82	5.9

**Table 8:** Comparison of different detection networks.

Models	*P*/%	*R*/%	mAP@0.5/%	FLOPs/G	Param/M	Weights/MB
SSD	64.1	58.1	68.6	207.5	54.68	78.8
Faster R-CNN	62.3	71.1	69.5	370.2	87.13	93.7
RT-DETR	71.4	73.7	70.7	125.7	41.95	86.1
YOLOv5s	73.2	78.0	76.5	21.3	9.42	19.2
YOLOv7x	71.8	75.2	75.1	23.2	6.03	12.3
YOLOv8s	86.3	80.0	87.5	28.5	11.13	22.5
YOLOv10s	79.3	69.1	74.4	21.4	7.22	16.6
YOLOv8s-Piglet (ours)	92.6	91.2	91.8	14.1	2.82	5.9

**Table 9:** Comparison of average inference times before and after deployment.

Deployment Device	Models	Inference Time/ms
Desktop Computer	YOLOv8s	19.7
	YOLOv8-Piglet	6.6
Jetson Orin NX	YOLOv8s	353.9
	YOLOv8-Piglet	163.2

## Data Availability

The data presented in this study are available on request from the corresponding author. (the data are not publicly available due to privacy).
